# Bone Cells in Birds Show Exceptional Surface Area, a Characteristic Tracing Back to Saurischian Dinosaurs of the Late Triassic

**DOI:** 10.1371/journal.pone.0119083

**Published:** 2015-04-01

**Authors:** John M. Rensberger, Ricardo N. Martínez

**Affiliations:** 1 Department of Earth and Space Sciences, University of Washington, Seattle, Washington, United States of America; 2 Museo de Ciencias Naturales, Universidad Nacional de San Juan, San Juan, Argentina; Université de Lyon - Université Jean Monnet, FRANCE

## Abstract

**Background:**

Dinosaurs are unique among terrestrial tetrapods in their body sizes, which range from less than 3 gm in hummingbirds to 70,000 kg or more in sauropods. Studies of the microstructure of bone tissue have indicated that large dinosaurs, once believed to be slow growing, attained maturity at rates comparable to or greater than those of large mammals. A number of structural criteria in bone tissue have been used to assess differences in rates of osteogenesis in extinct taxa, including counts of lines of arrested growth and the density of vascular canals.

**Methodology/Principal Findings:**

Here, we examine the density of the cytoplasmic surface of bone-producing cells, a feature which may set an upper limit to the rate of osteogenesis. Osteocyte lacunae and canaliculi, the cavities in bone containing osteocytes and their extensions, were measured in thin-sections of primary (woven and parallel fibered) bone in a diversity of tetrapods. The results indicate that bone cell surfaces are more densely organized in the Saurischia (extant birds, extinct Mesozoic Theropoda and Sauropodomorpha) than in other tetrapods, a result of denser branching of the cell extensions. The highest postnatal growth rates among extant tetrapods occur in modern birds, the only surviving saurischians, and the finding of exceptional cytoplasmic surface area of the cells that produce bone in this group suggests a relationship with bone growth rate. In support of this relationship is finding the lowest cell surface density among the saurischians examined in Dinornis, a member of a group of ratites that evolved in New Zealand in isolation from mammalian predators and show other evidence of lowered maturation rates.

## Introduction

### Structural Organization and Growth

Skeletal bone provides the framework supporting the body and the mechanism for locomotion, making its strength and rate of maturation vital to each individual. It has long been recognized that the rate at which bone tissue is formed varies in relationship to its structural organization [1], [2]. Woven bone, characterized by an irregular microstructural organization, is the fastest forming type of tissue, with rates of more than 4 μm per day, but sacrifices strength compared to slower forming tissues [3]. Lamellar tissue, which has a highly organized microstructure in which fiber directions change through successive layers, is the slowest forming type of tissue, with rates of less than 1 μm per day [4] but is the strongest type of bone. Parallel-fibered bone lacks the more complex organization of lamellar bone and forms at rates intermediate between those of woven and lamellar tissues [3].

At a higher level of structural organization, fibrolamellar (plexiform) bone [5]–[7] is a vascular based tissue that increases the diametric rate of bone growth by enlarging the surface areas on which new bone can simultaneously form. In this tissue, trabeculae of rapidly formed woven or parallel-fibered bone are extended outward from the growing surface, leaving unfilled spaces around the vascular canals which are subsequently filled with slower forming but stronger lamellar tissue to form primary osteons [8], [9]. The proportion of primary osteons in fibrolamellar bone also correlates with the tissue growth rate [10]. Fibrolamellar tissue is present in many large dinosaurs and mammals [2], [11], [12], [13].

At a finer level of organization, two microstructural features, random directionality of canaliculi in secondary osteons, resembling that in primary woven bone, and reduced abundance and quality of lamellar tissue have been found to be distinctive features of birds and small late Cretaceous theropods [14]. The reduced quantity and quality of lamellar tissue and the increased resemblance of the tissue in secondary osteons to woven tissue in those taxa suggests accelerated osteogenesis in these ontogenetically later forming bone tissues.

In a morphometric study of human tissue, greater numbers of canaliculi were counted on the walls of osteocyte lacunae in woven than in the more slowly forming lamellar bone [15] and another study of seven taxa (frog, chicken, rabbit, bovine, horse, dog and human) found greater numbers of canaliculi departing from osteocyte lacunae in woven than in the slower forming parallel-fibered bone [16].

**Fig 1:**
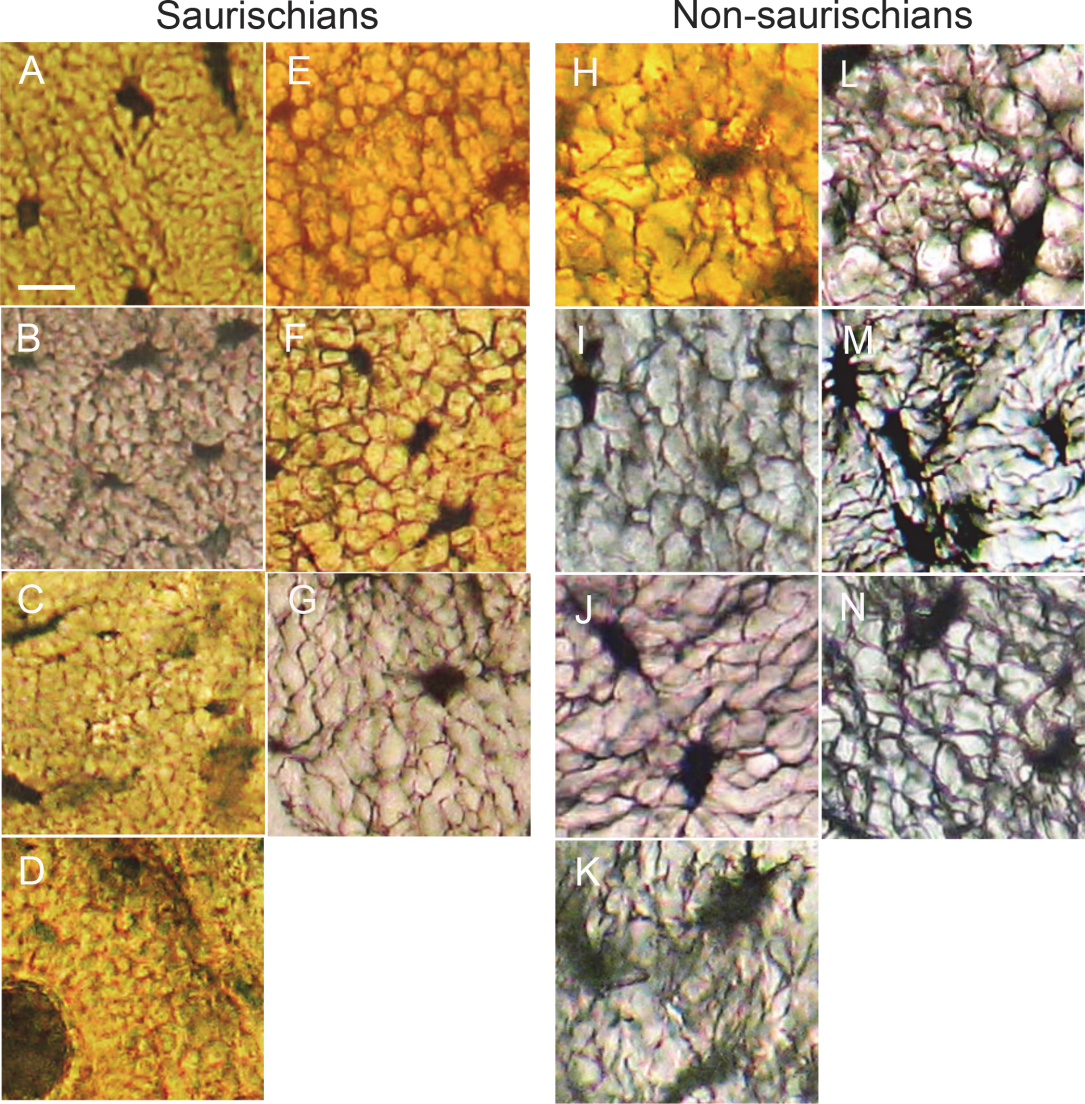
Osteocyte lacunae and canaliculi in woven cortical bone. (A) Neornithes (Recent great horned owl, *Bubo virginianus*) ulna. (B) Ornithomimidae (late Cretaceous theropod) metatarsal. (C) *Tyrannosaurus* (late Cretaceous theropod) phalanx. (D) Titanosauridae (late Cretaceous sauropod) limb. (E) *Adeopapposaurus* (early Jurassic basal sauropodomorph) rib. (F) *Herrerasaurus* (late Triassic basal theropod) neural spine. (G) *Dinornis* (sub-Recent ratite) tarsometatarsus. (H) Ornithischia (late Cretaceous hadrosaurid) ossified tendon. (I) Phytosauria (late Triassic aquatic archosaur) vertebra. (J) *Alligator* (Recent crocodylomorph archosaur) tibia. (K) Lacertilia (lizard, *Tupinambis*) humerus. (L) *Mammalia* (armadillo, *Dasypus*) femur. (M) *Mammalia* (prong-horn, *Antilocapra*) metatarsal. (N) Amphibia (frog, *Rana*) humerus. Images are shown at the same scale: 10 μm bar in (A).

A visual comparison of samples of woven and parallel-fibered bone tissues from a diversity of taxa (Fig. 1) suggests that canaliculi in three groups of saurischian dinosaurs, modern birds (A), non-avian theropods (B,C) and sauropodomorphs (D, E), are more densely organized than in non-saurischian taxa (H-N). Because canaliculi contain the cytoplasmic extensions of the cells which produced the bone tissue, a greater density of canaliculi may reflect a capacity for higher rates of osteogenesis. However, variation can be seen both within taxa and within individual samples (Fig. 1). To test the hypothesis that the cytoplasm of bone cells in primary tissue is more densely organized in saurischians than in other tetrapods, we measured in thin-section the perimeters of osteocyte lacunae, the lengths of canaliculi, the number of canalicular branching points and the total canalicular and lacunar perimeters (a correlate of the total cellular surface area) within sampled areas of equivalent size. To make the measurement process uniform, image pixel shading was converted to binary values (providing black canaliculi and lacunae on white backgrounds) using a uniform set of conversion parameters across all images, and measurements were made using an image processing algorithm (see Methods for details).

The measured samples encompass five mammalian genera, three lizard genera, a phytosaur, a crocodylomorph, five modern bird genera, two Cretaceous theropod dinosaurs, two late Triassic theropods, a late Cretaceous sauropod dinosaur (titanosaurid), a late Jurassic sauropod, an early Jurassic basal sauropodomorph, and two late Cretaceous ornithischian dinosaurs.

## Methods

Samples of fossil cortical bone tissue were obtained from collections in five museums (see below). Samples of Recent vertebrates were obtained from donated skeletons and road-kills. No living animals were sacrificed for this project.

### Thin-section preparation

Sections of cortical bone were cut at thicknesses of 1 to 2 mm and then ground to a thickness of 50 μm or less with an Ingram thin-section grinder. Digital images were taken with transmitted light at a microscope magnification of 400x and a camera resolution of 4.4 pixels per micron. Various postcranial elements were sampled in planes perpendicular to the long axis of the bone, creating transverse sections, and some were also sectioned parallel to the long axis (longitudinal sections). Transverse sections were used for measurements of osteocyte canalicular and lacunar dimensions because the canaliculi extend predominantly in that plane and perpendicular to the long axis of the bone, i.e. parallel to the predominant (radial) direction of growth. Areas measured were of identical size (45 μm x 45 μm) in regions lacking artifacts such as cracks and containing the best preserved canaliculi.

### Image preparation

**Fig 2:**
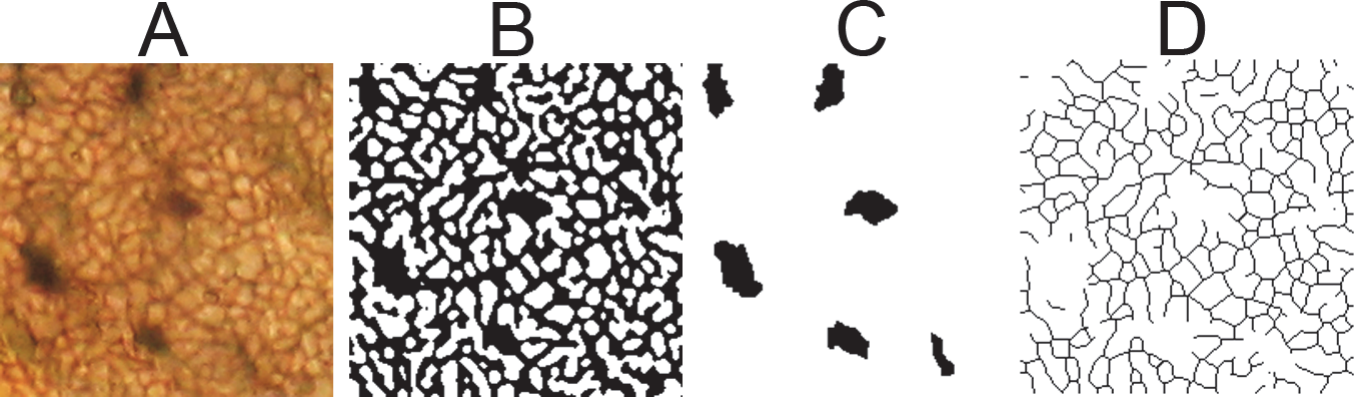
Sequence of image processing steps used to obtain binary osteocyte lacunar and canalicular images for measurement. (A) 400x microphotograph. (B) Binary image derived from (A) after the filtering sequence described in Methods. (C) Osteocyte lacunae extracted from (B). (D) Canaliculi remaining after extraction of lacunae and conversion to 1 pixel wide skeletons. The filtering procedure was applied identically across all samples.

To make structural boundaries unambiguous and measurements uniform, each image was subjected to the following sequence of image processing filters and their settings in Adobe Photoshop in order to equalize image contrast and render osteocyte lacunae and canaliculi as black objects on white backgrounds (Fig. 2):

1. High Pass: radius set to 4.6 pixels.
2. Levels: left and right pointers set to left and right margins, respectively, of the image density distribution and the central pointer set to the central modal value.
3. Threshold: Level set to the central (default) value: 128
4. To remove noise artifacts and produce an image like that of Fig. 2B, each thresholded image was subjected to the following sequence of filters and settings from the Image Processing Toolkit in Adobe Photoshop:
  - Dilate (parameters: 4, 2)
  - Erode (parameters: 3, 1)

Cracks, which can be induced when thin-sections are ground by manually applying pressure on the sample, were not a problem because the sections in this study were prepared with the Ingram thin-section grinder in which the section is advanced toward the grinding wheel at a rate determined by a finely adjustable mechanical control. In the instance in which a crack was introduced, that region was omitted from measurement.

### Measurement of canalicular density and lacunar cross-sectional area

Images of osteocyte lacunae were extracted from the binary image (Fig. 2B) using the Photoshop Lasso tool to encircle the black lacunae on white backgrounds and saved, producing two images, one containing just the lacunae (Fig. 2C) and another containing only canaliculi. The canalicular image was subjected to the Image Processing Toolkit filter "Skeletonize" to reduce the canaliculi to widths of 1 pixel (Fig. 2D). The lacunar image and the canalicular image were then each measured separately using the IPT algorithm "Measure All" to provide the osteocyte lacunar perimeters, the number of canalicular branch points and the lengths of the segments between branch points. For each sample, the sum of the lacunar perimeters was added to twice the total canalicular length (the latter approximating the canalicular perimeter) to provide a correlate of the cytoplasmic surface area in the sample. Although osteocyte lacunae are slightly larger than the contained osteocyte, this is characteristic of mature bone in all of the taxa examined and does not impair a uniform comparison of differences. Significances of differences in the frequency distributions were calculated with Fisher’s Exact test (1-tailed).

Most measurements of osteocyte lacuna dimensions (length, width or area) in thin-section will be smaller than the actual cell dimension because each lacuna will be intercepted by the plane of the thin-section at a position which will vary relative to the lacunar center, and the lengths of the minor axes in an individual cell are often different, although with a small standard deviation [17]. Consequently, only the largest lacuna measured in a sample was used to compare the differences among taxa.

### Institutional abbreviations

NMMNH: New Mexico Museum of Natural History and Science, Albuquerque, New Mexico.

PVSJ: Instituto y Museo de Ciencias Naturales, Universidad Nacional de San Juan, San Juan, Argentina.

ROM: Royal Ontario Museum,Toronto, Ontario.

UCM: University of Colorado Museum of Natural History, Boulder, Colorado.

UWBM: Burke Museum of Natural History and Culture, University of Washington, Seattle, Washington.

### Taxa and bones measured

Neornithes:

*Buteo jamaicensis* UWBM 82968 humerus (Recent, Seattle).

*Bubo virginianus* UWBM 86698 ulna (Recent, Seattle).

*Haliaeetus leucocephalus* UWBM 83090 tarsometatarsus (Recent, Olympic Peninsula, Washington).

*Cygnus columbianus* UWBM 82963 tibiotarsus (Recent, Summer Lake, Oregon).

*Mergus merganser* UWBM 87129 tibiotarsus (Recent, Seattle).

*Dinornis* sp. UWBM 27558 tarsometatarsus (subRecent, New Zealand).

Theropoda:

*Tyrannosaurus* ROM 1799 phalanx III (late Cretaceous, Canada).

Ornithomimid ROM 47423 metatarsal (late Cretaceous, Canada).

Ornithomimid UWBM 31778 phalanx, (late Cretaceous, Montana, 47° 40' N, 107° 10' W).

*Coelophysis* baueri NMMNH L-3115 vertebra (late Triassic, New Mexico).

*Herrerasaurus ishigualastensis* PVSJ 373 neural spine and zygapophysis (late Triassic Ischigualasto Formation, San Juan, Argentina).

Sauropodomorpha:

Diplodocidae UWBM 87927 femur (locality unknown).

Titanosauria limb (late Cretaceous, Lameta Fm. India).

*Adeopapposaurus mognai*, PVSJ 569 r**ib** (Jurassic Upper Pelitic Member Canon del Colorado Formation, southwestern Sierra de Mogna, San Juan.

Ornithischia:

Hadrosauridae UWBM 87454 rib (47° 33‘ N, 107° 10’, late Cretaceous Hell Creek Formation, Montana).

Hadrosauridae UWBM 76357 ossified tendon (47° 33‘ N, 107° 10’ W, late Cretaceous Hell Creek Formation, Montana).

*Triceratops* UWBM 76508 horn (47° 32’ N, 106° 59‘ W, late Cretaceous Hell Creek Formation, Montana).

Crurotarsi:

Phytosauria UCM 11463 vertebra (Late Triassic, U. S).

*Alligator mississippiensis* UWBM 84007 tibia (Recent, Florida).

Mammalia:

*Dasypus novemcinctus* UWBM 86699 femur (Recent, southeastern U. S.).

*Didelphis virginiana* UWBM 83756 ulna (Recent, Seattle, Washington).

*Capromys piloride*s UWBM 83815 humerus (Recent, locality unknown).

*Canis latrans* UWBM 27411 tibia (Recent, locality unknown).

*Puma concolor* UWBM 83893 metacarpal (Recent, Seattle, Washington).

*Antilocapra americana* UWBM 84006 metatarsal (Recent, SW Wyoming).

Lacertilia:

*Tupinambis teguixin* UWBM 83798 humerus (Recent, locality unknown).

*Tarentola* mauritanica vertebra (Recent, locality unknown).

*Heloderma horridum* vertebra (Recent, locality unknown).

## Results

### Density of cytoplasmic processes

**Fig 3:**
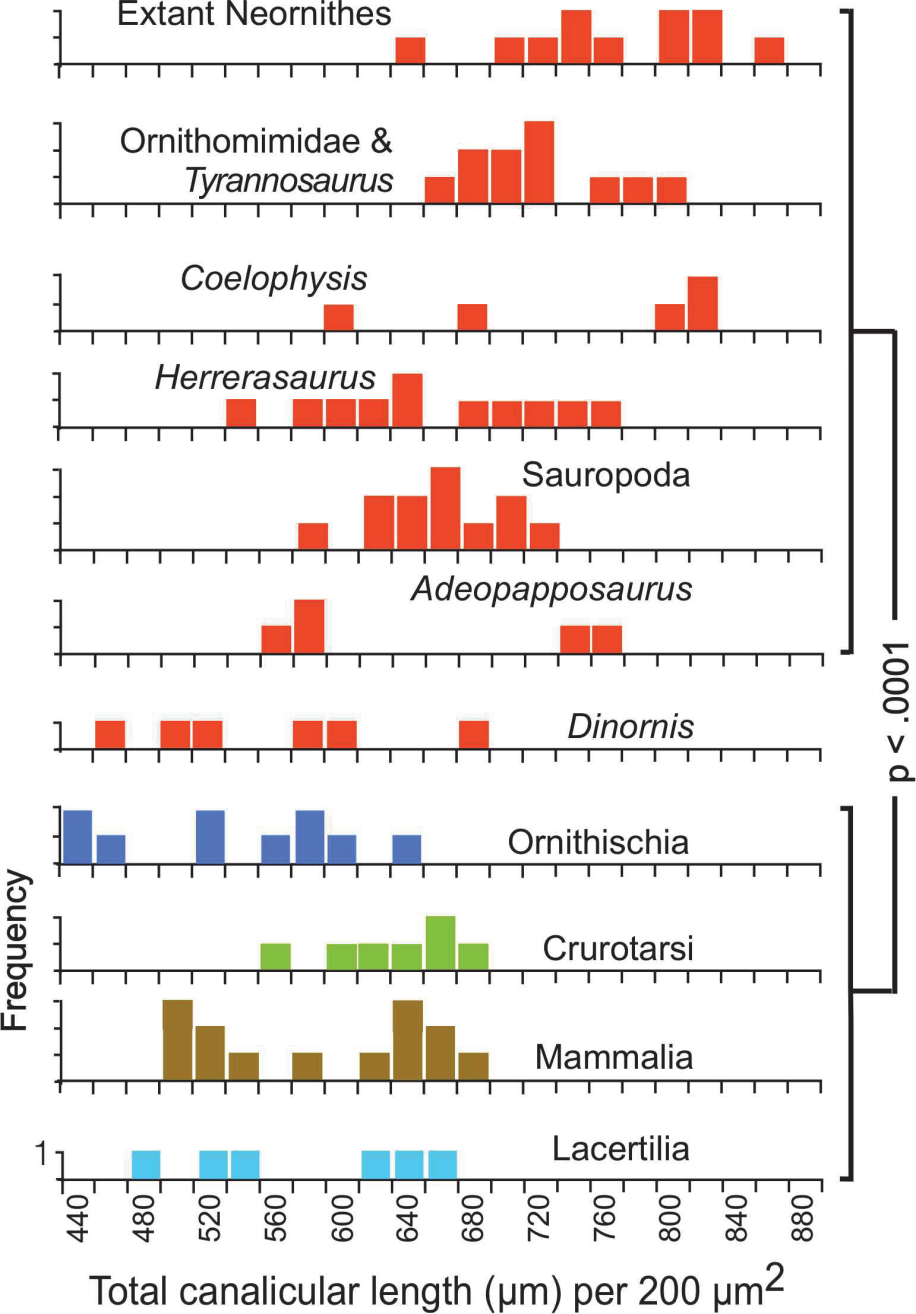
Total lengths of canaliculi per sample. Each sample is 770,884 μm^2^ in area; null probabilities were calculated with Fisher's Exact test; red frequency distributions identify Saurischia in this and subsequent figures. Original measurements for Figs. 3, 4 and 5 are listed in S1 Table.

**Fig 4:**
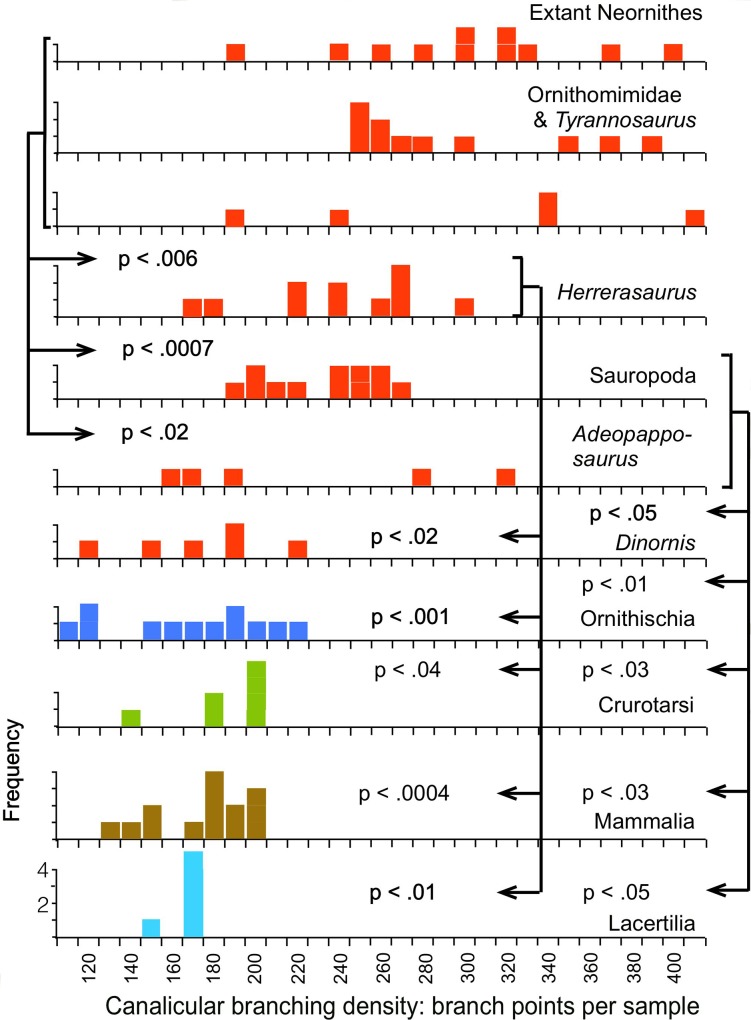
Total number of canalicular branching points per sample. See also legend for Fig. 3.

The measurements show that the total lengths of the canaliculi within samples of equivalent area (Fig. 3) are greater in the saurischians (Neornithes, Ornithomimidae, *Tyrannosaurus*, *Coelophysis*, *Herrerasaurus*, Sauropoda, and *Adeopapposaurus)* than in the non-saurischians (Ornithischia, Crurotarsi, Lacertilia and Mammalia), a result indicating greater canalicular density. Among the saurischians, the greatest canalicular lengths were found in the modern birds and late Mesozoic theropods, and were slightly greater in the birds than in the other theropods (p = 0.043). These elevated canalicular lengths result from more frequent branching, as shown by the greater density of branch points (Fig. 4).

Although canalicular branching densities in the sauropodomorphs and the early theropod *Herrerasaurus* are somewhat lower than in the birds and the Cretaceous theropods, they are nevertheless significantly higher than those in the ornithischian dinosaurs, alligator, phytosaur, lizards or mammals (Fig. 4). A highly branched appearance of osteocytes observed in the late Triassic theropod *Syntarsus* [18] likely reflects the same pattern. Only one of the saurischians sampled, *Dinornis*, showed canalicular densities as low as those in the non-saurischians; the implications of this are discussed below.

**Fig 5:**
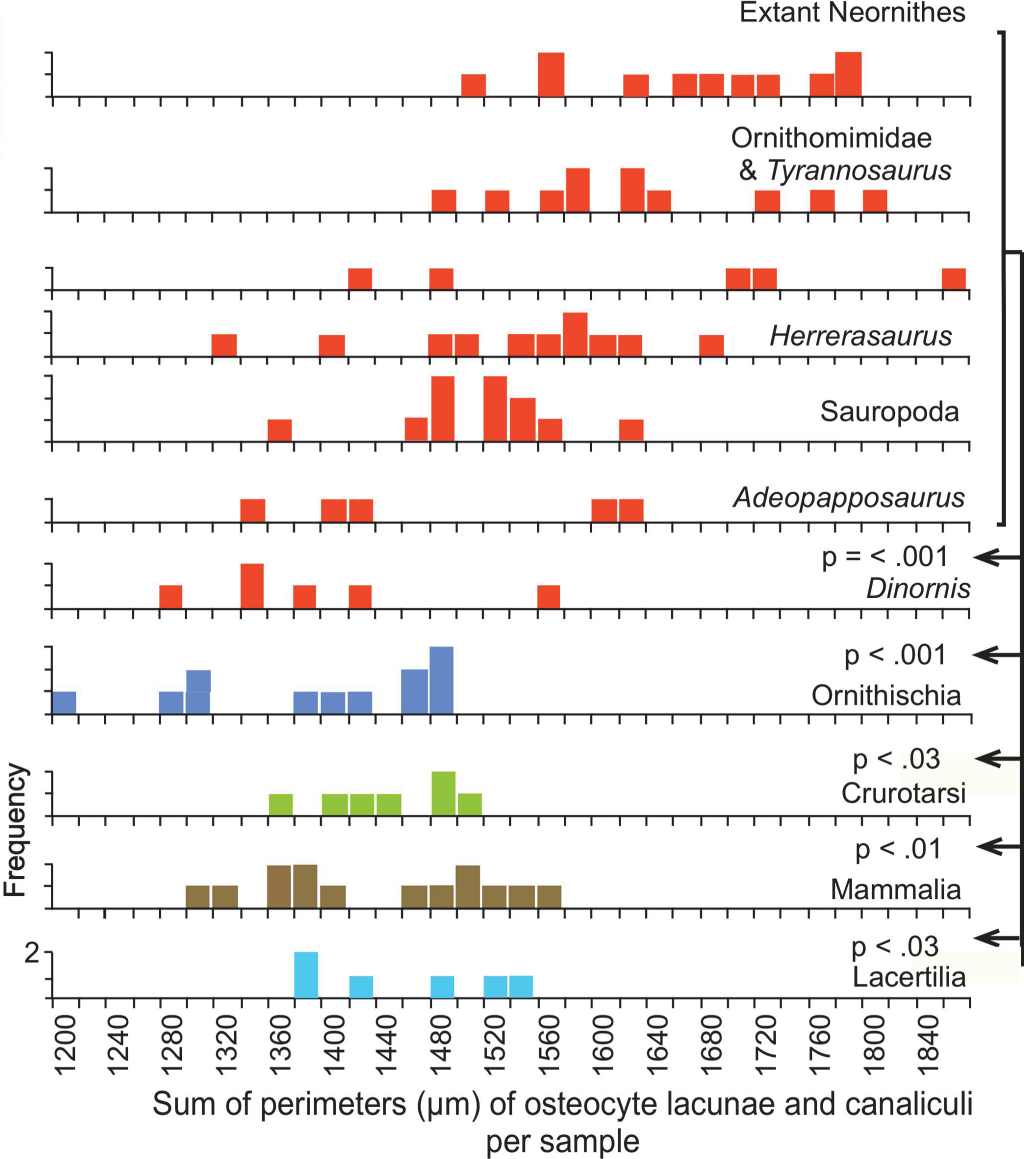
Combined perimeters of lacunae and canaliculi per sample. See also legend for Fig. 3.

Because the osteocyte lacunae and canaliculi enclose the cell bodies and their cytoplasmic extensions, the perimeters of the lacunae and the canaliculi measured in thin-section represent a correlate of the total surface area of the cell cytoplasm. The measured perimeters in saurischian thin-sections are significantly greater when compared across equivalent areas than those in any of the non-saurischian taxa (Fig. 5), indicating that the cell surface areas are densest in the Saurischia.

### Relationship of cytoplasmic processes with osteogenesis

Osteoblasts produce bone tissue during the interval in which they are extending cytoplasmic processes. In a study of the periosteal bone of young rabbits, it took approximately three days for an osteoblast to become an embedded osteocyte, during which time it manufactured two to three times its own volume of matrix [19]. The shape of the osteoblast changed during this transition, with cytoplasmic processes emerging from the cell as new bone tissue was being formed. Cell processes have been observed extending deep into abundant collagen fibers on the osteoid side of the cell while the other cell surface had fewer cell processes and fewer collagen fibers [20]. In a study of bone cells in the chick embryo [21], cytoplasmic processes were observed initially emerging from the side of the osteoblast facing the advancing mineralization front; where the front had passed a cell, the cytoplasmic protrusions were seen emerging on the opposite side of the cell, again facing the mineralization front. In another study, using the cell line MLO-A5 bearing markers of late osteoblasts [22], at 3 to 4 days of culture 20 to 50 nm spheres containing calcium and phosphorus were observed around and budding from developing cytoplasmic processes and by 5 to 6 days the calcified spheres were continuing to enlarge and engulf the collagen fibril network. Casein kinase II, believed to be responsible for the phosphorylation of the matrix proteins necessary for mineralization, is produced in large amounts by osteoid-osteocytes and not by osteoblasts [23]. For cells in general, differences in volume or surface area are believed to affect metabolic flux, biosynthetic capacity and nutrient exchange [24]. These observations show a close relationship in the timing of the formation of osteoblast processes and production of the components of new bone tissue and suggest that a greater density of the cytoplasmic surface areas of the bone producing cells caused by greater density of branching of their processes will elevate the maximum rate of osteogenesis.

A relationship between the high canalicular branching density in the saurischians and their maximum osteogenetic rate is also supported by the high rates of growth that have been observed in the only surviving saurischians, birds, which exhibited the highest branching densities we have measured. The postnatal growth rates of extant birds have been found to be almost five times those of mammals on average [25] and the highest measured rates of radial bone growth have been found in birds [11], [13], [26]. Rates of osteogenesis of up to 40 μm/day have been measured in the mallard *Anas platyrhynchos* [11], 80 μm per day in the emu *Dromaius novaehollandiae* [13] and 171 μm/day in king penguin chicks (*Aptenodytes patagonicus*), the highest measured rate known to date [26].

Another avenue to increased cell surface area could be through an increase in density of the cell bodies. Osteocyte lacunar density was observed to be greater in parallel-fibered than in slower forming lamellar bone in frog, sheep, dog, bovine, horse and humans [27].

### Well-developed lamellar tissue retained in early saurischians

**Fig 6:**
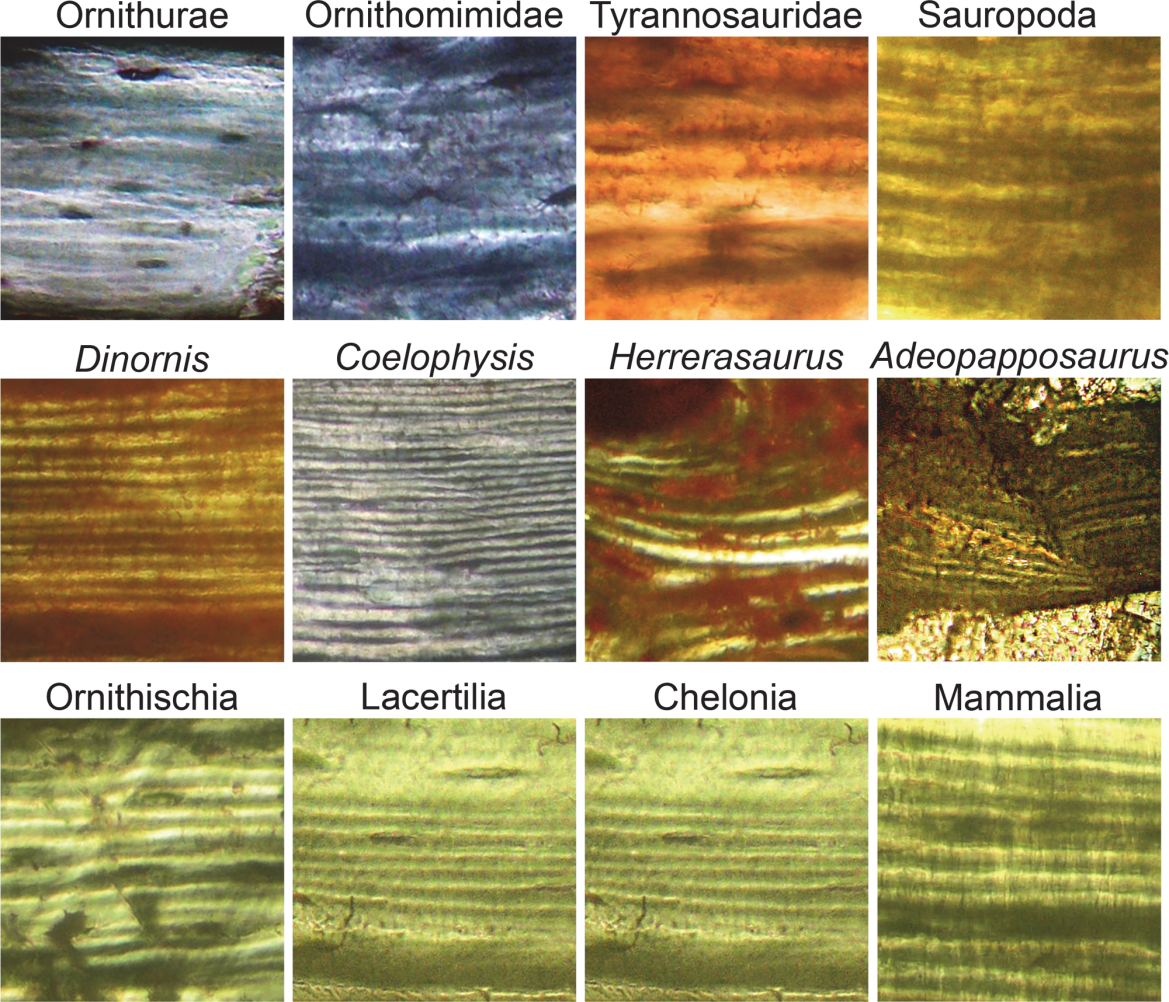
Differences in quality (boundary definition and continuity) of bone lamellae. Top row—late Cretaceous to Recent saurischians (poorly formed lamellae). Left to right: Ornithurae (*Phalacrocorax*, Recent); Ornithomimidae (small theropod, late Cretaceous); *Tyrannosaurus* (large theropod, late Cretaceous); Sauropoda (titanosaurid, late Cretaceous). Middle row—Saurischians with well-formed distinct lamellae. *Dinornis* (Moa, sub-Recent); *Coelophysis* (small theropod, late Triassic); *Herrerasaurus* (basal theropod, late Triassic); *Adeopapposaurus* (basal sauropodomorph, early Jurassic). Bottom row—Non-saurischians. Ornithischia (hadrosaurid, late Cretaceous); Lacertilia (*Tupinambis*, lizard, Recent); Chelonia (testudinid, late Cretaceous); Mammalia (*Puma*, Recent).

The appearance of increased canalicular density in the late Triassic *Herrerasaurus* and the early Jurassic *Adeopapposaurus* suggests that increased rates of osteogenesis began early in the saurischian phylogeny in both theropods and sauropodomorphs. This acceleration in development of the faster forming types of tissue appears to have extended to slower forming tissue (lamellar tissue) later in the Mesozoic, when irregular lamellae (Fig. 6) became characteristic of saurischians [14].

In modern birds and small late Cretaceous theropods, lamellar tissue has been observed to be less abundant and, where present, individual lamellae to be more poorly defined than in mammals and other non-saurischians [14]. We now find that larger saurischians, *Tyrannosaurus* and sauropods, also typically exhibit poorly defined and discontinuous lamellae (Fig. 6).

There is a relationship between the degree of fiber organization in bone tissue and the rate of its formation. Collagen fibers in woven bone, which forms at more than 4 μm per day, are oriented almost randomly [4]. Lamellar bone, which forms much more slowly [3]. consists of finely superposed layers in which the fiber direction has a much more structured organization. This association in modern taxa of rate of tissue formation with the degree of fiber organization suggests that the decrease in organization of secondary lamellar tissue in later saurischians is an indication of an extension of an increased pace of development that appeared in the primary tissue of the early saurischians to secondary tissue later in their phylogeny.

### Loss of saurischian microstructural characteristics in *Dinornis*

Attainment of reproductive maturity was delayed in the moa compared to all extant birds [28]. Although juvenile growth in the giant moa *Dinornis* was accelerated in order to reach great size, lines of arrested growth are seen in the middle cortex, as in members of the smaller moa family Emeidae [28], a condition rare in extant ornithurine birds [29], [30]. Our measurements show that the densities of canaliculi and their branch points and the total cytoplasmic surface area in *Dinornis* are significantly lower than in extant birds and non-avian saurischians and closely resemble the tetrapod plesiomorphic condition seen in mammals and other non-saurischians (Figs. 3–5). In addition, unlike extant birds and saurischians of the later Mesozoic, *Dinornis* shows well-developed lamellar tissue in which the borders of individual lamellae are better defined and extend over greater distances than in other saurischians (Fig. 6), again a suggestion of slower osteogenesis.

## Discussion

### Effect of reduced cell size

**Fig 7:**
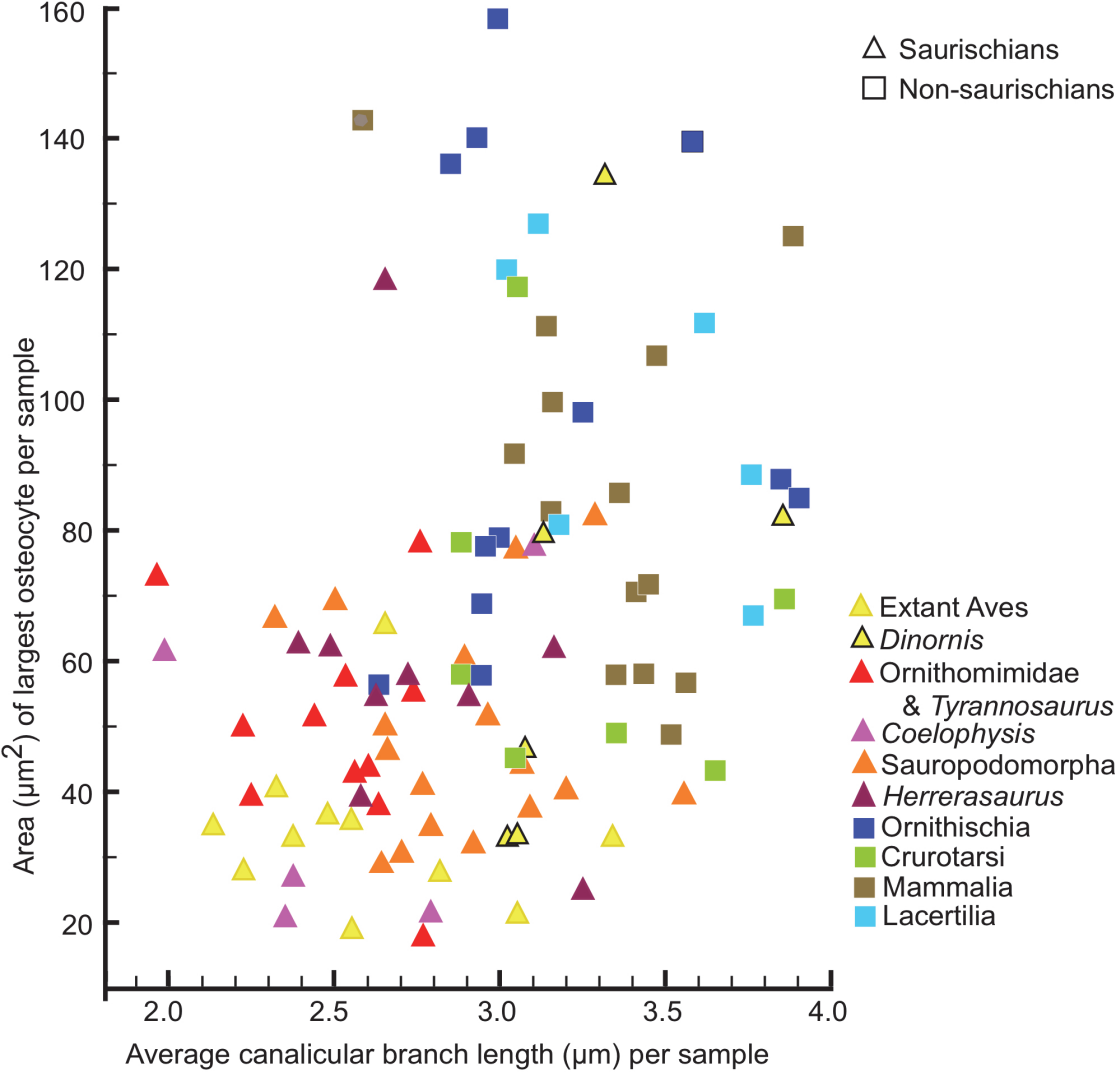
Relationship of osteocyte lacuna area and canalicular branch length. Osteocyte lacuna areas are shown for only the largest in each sample because the plane of a thin-section will pass through individual lacunae at widely varying distances from their centers. Branch length is the distance along the canaliculus between adjacent branching points. Note that there is no correlation within either the saurischians or the non-saurischians between osteocyte lacuna area and branch length, but the saurischians are distinguished by shorter canalicular branch lengths and smaller lacuna size.

The genome in birds is small, with only 10% composed of repeat elements compared to 40–50% in mammals [31], allowing smaller cell size. Measurement of the length and width of osteocyte lacunae has recently shown that the bone cells in birds and non-avian saurischian dinosaurs are smaller than in other tetrapods, including ornithischian dinosaurs and mammals [32], and our measurements confirm this (Fig. 7).

It may have been this smaller cell size that initiated the increased cytoplasmic branching density in the saurischians because a smaller size constrains the cell surface area by limiting the number of cytoplasmic processes that can emerge around the cell's circumference and increases the intercellular space. The filling of the larger cellular interspaces with more densely branched cytoplasmic processes in the saurischians results in a greater density of cell surfaces than is attainable in taxa with larger cell bodies

**Fig 8:**
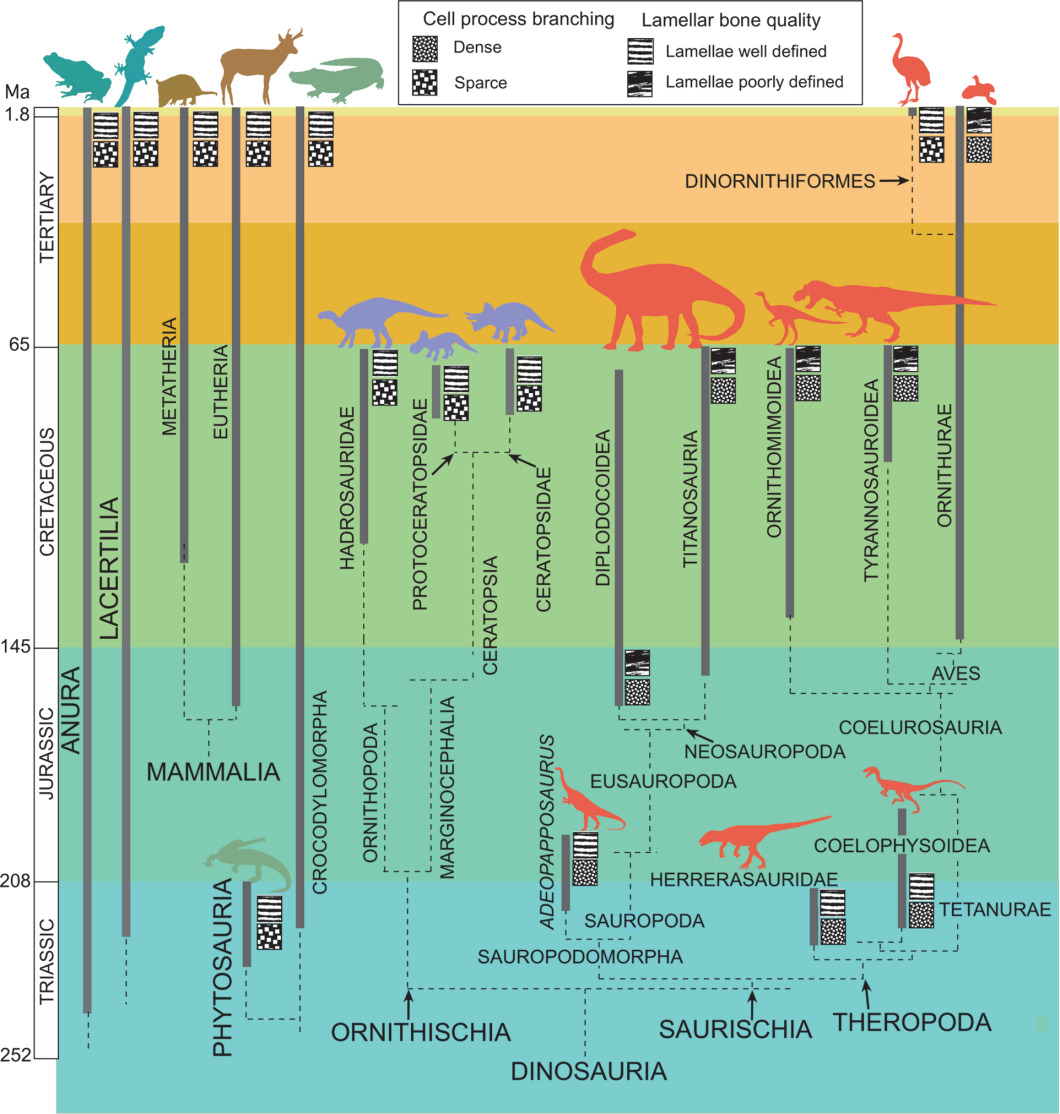
Phyletic differences in cytoplasmic density and lamellar quality. Exceptionally dense branching of the osteocyte cytoplasmic processes characterizes all of the sampled saurischians except the moa *Dinornis*. Note that reduction of lamellar quality had not yet appeared in the earliest theropod and sauropodomorph members. Phyletic relationships from [33]. [34]

Although elevated density of canalicular branching appeared in the primary tissue of saurischians as early as the late Triassic (in *Herrerasaurus* and *Coelophysis)*, the retention in those taxa of well-developed lamellar tissue, which is stronger but slower forming, suggests selection was initially more intense for accelerated attainment of size and locomotor ability than for skeletal reinforcement. It appears then that early in the saurischian phylogeny (Fig. 8) the first structural modification was an increase in density of the cell processes in woven and parallel fibered bone, a change that enabled acceleration in the maturation of the skeletal framework. Then, in later Mesozoic saurischians, the acceleration in osteogenesis became extended to lamellar tissue, which forms later in the individual development and serves to strengthen the skeleton. This phyletic delay in acquisition of faster forming but poorer quality lamellar bone in saurischians is consistent with the importance of the role of lamellar tissue in strengthening the skeletal framework. The eventual reduction in lamellar quality indicates an extension of selection for more rapid osteogenesis beyond tissues that build the skeleton to those that reinforce and repair the mature skeleton.

The phyletic reduction of canalicular density in *Dinornis* and the accompanying reappearance of strong lamellar bone, both of which are consistent with slower tissue formation, may indicate that the elevated canalicular density and faster growing type of lamellar tissue was maintained in other Saurischia through their long phyletic history by selection for rapid maturation in the presence of predation pressure. In *Dinornis*, in which that selection was relaxed, the tissues returned to their plesiomorphic structure.

Ornithischian dinosaurs lacked the elevated canalicular branching density and irregular lamellar tissue of saurischians but, like larger mammals, they utilized fibrolamellar organization to accelerate radial bone growth and reach exceptional size. However, their largest members didn't attain the sizes of the largest saurischians, and this suggests that the canalicular branching density in saurischians had a role in their attainment of gigantism.

### Broad distribution of elevated canalicular branching density in the saurischian skeleton

Different rates of radial growth have been shown to occur in different bones and within different regions of the same bone [11], [26]. Although the samples in this study included a wide range of different skeletal elements, from rib to proximal and distal limb bones, we did not observe differences in canalicular branching patterns between different bone elements. The rate of growth at any given time in an individual's ontogeny is likely constrained by many factors, including stage of development [35], [36], accessibility of nutrients [3], [26], [37], [38] and the immediacy of required function in the specific skeletal element [13], [39]. The elevated cytoplasmic branching density in the Saurischia appears to be a feature that is broadly distributed through the skeletal tissues. Its effect on growth rate would be to elevate the maximum rate for osteogenesis. The actual rate of osteogenesis at a specific time and location in the skeleton would be modulated by other factors, such as availability of nutrients, local abundance of vascular canals, etc.

Recent measurement of osteocyte lacunar dimensions in birds [40] showed that taxa with lower growth rates tend to have larger lacunae, in contrast with other studies which found that larger osteoblasts secrete more osteoid [20], [23], [24] and a study showing that larger cells are found in femora of animals with higher growth rates [41]. Our results show that cell bodies of smaller diameter allow larger cell surface areas if the cell interspaces are filled with a greater abundance of densely branching cell processes. Future studies in which cell mass is measured as the sum of the lacunar volume plus that of the cell processes may resolve this conflict.

It was recently suggested [42] that because secondary bone is derived from dynamic osteogenesis and therefore characterized by a strongly organized structure, the irregular directionality of canaliculi that had been observed in the secondary osteons of extant birds [14] must have resulted from local differences in cell orientation which may have been caused by different patterns of loading. However, the random canalicular directions result from the densely divergent branching of the canaliculi, as shown here, rather than from differences in cell orientation and occur too extensively throughout the skeletal elements to be the result of special loading conditions. Furthermore, the planes of sections were standardized in the study finding canalicular irregularity in saurischian secondary osteons [14].

### Stress detection

An increasingly supported hypothesis for the function of the osteocyte-canalicular networks after the cells and their processes are fully enclosed in mineralized bone is that they carry signals of differences in electrical potential (SGPs) generated when the bone is loaded and that these signals control the remodeling of bone [43]–[46]. The osteocytes themselves have been presumed to be the mechanical sensors, but *in vivo* tissue strains that have been observed in bone [47], [48] have been found to be much smaller than the strain levels needed to activate cell signaling in cultures [49].

This problem now may be resolved. Fluid flow is induced in the pericellular space of the canaliculi by mechanical loading of the bone [45], [47], [50]–[52]. The cytoplasmic processes have been observed to be centered in the canaliculus by transverse elements that cross the pericellular space, tethering the processes to the canalicular wall by β3 integrins [53], [54]. Fluid flow acting on the tethering fibers should be two orders of magnitude greater than the in-bone strains that induce the fluid flow, and would be high enough to elicit intracellular biochemical responses [54]. In that context the denser, more highly branched cytoplasmic networks in the Saurischia could have the effect of increasing the resolution of this strain detection system. This would be especially advantageous in fragile, thin-walled saurischian bones. Osteogenesis and strain detection are not mutually exclusive functional hypotheses for elevated canalicular density because they would apply at different stages of development, with strain detection functioning after the tissue is mineralized.

In mammals lamellar bone has been found to be an incremental tissue, with each lamella formed in a species-specific time frequency corresponding with the striae of Retzius formation in enamel [55]. The poor distinction of individual lamellae in birds and Mesozoic saurischians may reflect an accelerated pace of formation of lamellae that overrides this natural rhythm.

## Conclusions

Saurischian dinosaurs have, in their fastest forming types of bone tissue, the most densely organized bone cell cytoplasm of any of the tetrapod groups examined. This higher density results from more profuse branching of the cell processes and is first seen in late Triassic basal saurischians.

This increased density appears to have been initiated by reduction in the size of the cell bodies because smaller cell perimeters limit the number of branches that can emerge directly from the cell body. However, the increase in branching density did not merely maintain the cellular surface density of earlier tetrapods but exceeded it because the smaller sizes of the cell bodies created larger intercellular spaces containing the more densely branching processes. The resulting higher ratio of cell surface to bone volume elevated a constraint limiting the rate of secretion of the constituents of new bone tissue from the osteoblasts and may have enabled higher rates of osteogenesis when other factors, such as availability of nutrients, are favorable, or, in a phylogenetic context, when an elevated rate of maturation of an individual bone element becomes advantageous.

Although Increased cytoplasmic surface density is present in the late Triassic basal saurischian *Herrerasaurus* and the early Jurassic sauropodomorph *Adeopapposaurus*, those early taxa still retained the strong, well defined lamellar tissue characteristic of non-saurischians. It is in later saurischians that lamellae become more irregular and poorly defined, indicating that accelerated osteogenesis appeared first in tissues building the skeleton and phylogenetically later extended to tissues involved in bone reinforcement and repair.

That the elevated density of the cytoplasmic surface area is related to the rate of osteogenesis is supported by (1) occurrence of the highest canalicular branching density in birds, which have the highest rates of radial bone growth among modern tetrapods; (2) reduction of the high canalicular branching density and return to more regular lamellar tissue in *Dinornis*, a member of a group of birds that had a slower maturation rate in an environment with lowered predation pressure; and (3) attainment in the Saurischia of the greatest body sizes known among terrestrial carnivores and herbivores.

After bone is fully formed in saurischians, the elevated density of the canalicular network may also increase the sensitivity and resolution of the strain detection mechanism that is believed to guide the bone modeling and remodeling processes.

## Supporting Information

S1 Table Measurements. (DOC)
