# Correction: Bone Cells in Birds Show Exceptional Surface Area, a Characteristic Tracing Back to Saurischian Dinosaurs of the Late Triassic

**DOI:** 10.1371/journal.pone.0127373

**Published:** 2015-05-01

**Authors:** John M. Rensberger, Ricardo N. Martínez

There is an error in Fig 3, “Total lengths of canaliculi per sample.” Please see the corrected Fig 3 and its legend here.

**Fig 3:**
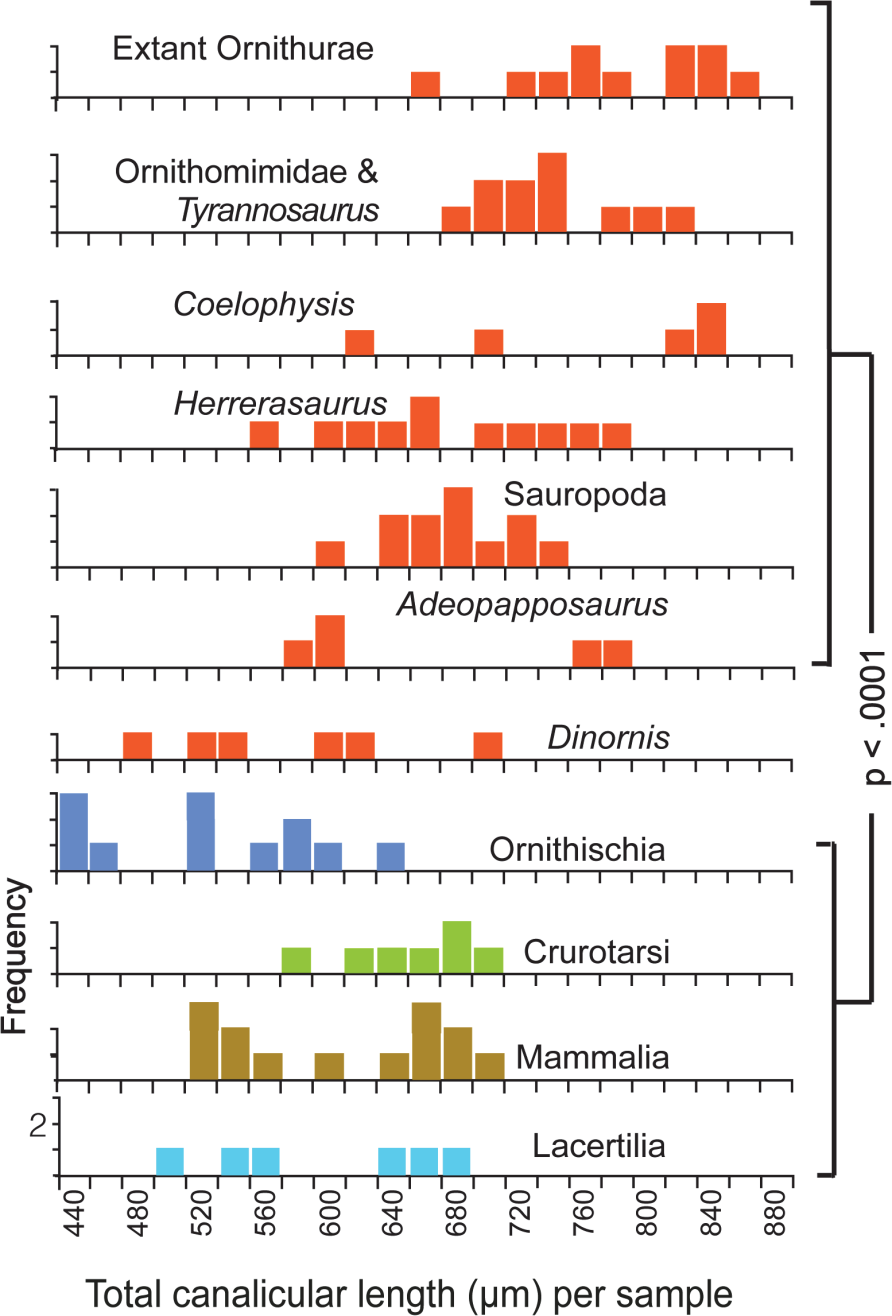
Total lengths of canaliculi per sample. Total lengths of canaliculi per sample. Each sample is 2025 μm2 in area; null probabilities were calculated with Fisher's Exact test; red frequency distributions identify Saurischia in this and subsequent figures. Original measurements for Figs 3, 4 and 5 are listed in S1 Table.

There is an error in S1 Table. Please view the correct S1 Table below.

## Supporting Information

S1 Table Measurements. (DOC)
