# Supplementary material for: Bone Cells in Birds Show Exceptional Surface Area, a Characteristic Tracing Back to Saurischian Dinosaurs of the Late Triassic
Source: PLoS One. 2015 Apr 1;10(4):e0119083. doi: 10.1371/journal.pone.0119083 (PMC4382344; doi:10.1371/journal.pone.0119083)
Supplement: S1 Table — (DOC) [file pone.0119083.s001.doc]

| Sample | Description | Total | Largest | Total | Max. | Total | Total | Total | Av. |  |
| --- | --- | --- | --- | --- | --- | --- | --- | --- | --- | --- |
|  |  | Lac | Lac | Lac | Lac | Can | Can | Lac + | Can |  |
|  |  | Area | Area | Peri | Peri | Lgth | Brs | Can Peri | Lgth |  |
|  |  | µm^2^ | µm^2^ | µm | µm | µm |  | µm | µm |  |
| **Extant Neornithes** | |  |  |  |  |  |  |  |  |  |
| 4738 Area1 | Buteo HUM | 131.35 | 40.50 | 132.81 | 33.26 | 715.99 | 308 | 1564.79 | 2.32 |  |
| 0013 Area1 | Buteo FEM | 116.32 | 28.94 | 124.57 | 24.33 | 835.96 | 376 | 1796.49 | 2.22 |  |
| 0013 Area3 | Buteo FEM | 116.73 | 36.92 | 102.99 | 31.93 | 807.46 | 327 | 1717.91 | 2.47 |  |
| 4806 Area1 | Buteo TBT | 53.07 | 35.23 | 64.01 | 30.25 | 860.49 | 403 | 1784.99 | 2.14 |  |
| 4806 Area2 | Buteo TBT | 119.85 | 33.44 | 118.87 | 25.02 | 804.31 | 337 | 1727.48 | 2.39 |  |
| 4806 Area3 | Buteo TBT | 85.95 | 27.56 | 101.86 | 27.09 | 734.27 | 261 | 1570.40 | 2.81 |  |
| 0191 Area1 | Bubo ULN | 145.87 | 65.60 | 131.11 | 45.15 | 751.26 | 283 | 1633.62 | 2.65 |  |
| 4736 Area1 | Haliaeetus TMT | 130.92 | 35.36 | 144.55 | 37.86 | 768.61 | 302 | 1681.78 | 2.55 |  |
| 0193 Area1 | Cygnus TBT | 194.48 | 32.06 | 182.77 | 27.39 | 658.92 | 196 | 1500.60 | 3.36 |  |
| 4772 Area1 | Mergus TBT | 122.15 | 21.32 | 169.01 | 21.23 | 755.17 | 248 | 1679.36 | 3.05 |  |
| 4772 Area2 | Mergus TBT | 87.87 | 18.84 | 110.99 | 23.39 | 825.92 | 321 | 1762.83 | 2.57 |  |
|  |  |  |  |  |  |  |  |  |  |  |
| **Ornithomimidae &Tyrannosaurus** | |  |  |  |  |  |  |  |  |  |
| 0349 Area2 | Tyrannosaurus PHL | 164.23 | 78.02 | 160.50 | 81.88 | 710.99 | 258 | 1582.48 | 2.76 |  |
| 018 Area1 | Tyrannosaurus PHL | 198.39 | 55.07 | 177.78 | 46.49 | 727.26 | 265 | 1632.29 | 2.74 |  |
| 018 Area2 | Tyrannosaurus PHL | 284.54 | 44.33 | 248.67 | 37.30 | 664.73 | 256 | 1578.14 | 2.60 |  |
| 019 Area1 | Tyrannosaurus PHL | 189.59 | 57.83 | 196.89 | 60.67 | 698.46 | 277 | 1593.82 | 2.52 |  |
| 4434 Area2 | Ornithomimidae PHL | 83.96 | 18.05 | 104.44 | 20.40 | 696.91 | 251 | 1498.27 | 2.78 |  |
| 4434 Area3 | Ornithomimidae PHL | 117.50 | 38.14 | 127.30 | 37.64 | 702.87 | 269 | 1533.04 | 2.61 |  |
| 4825 Area1 | Ornithomimidae MTT | 113.82 | 39.57 | 139.35 | 43.06 | 790.35 | 352 | 1720.04 | 2.25 |  |
| 4825 Area2 | Ornithomimidae MTT | 242.31 | 73.73 | 246.98 | 55.71 | 776.86 | 397 | 1800.70 | 1.96 |  |
| 4825 Area3 | Ornithomimidae MTT | 151.86 | 51.18 | 165.99 | 35.82 | 738.68 | 303 | 1643.35 | 2.44 |  |
| 4825 Area4 | Ornithomimidae MTT | 127.01 | 50.31 | 135.21 | 33.64 | 816.93 | 370 | 1769.07 | 2.21 |  |
| 4825 Area5 | Ornithomimidae MTT | 162.44 | 43.46 | 163.21 | 34.43 | 736.32 | 287 | 1635.86 | 2.57 |  |
|  |  |  |  |  |  |  |  |  |  |  |
| **Coelophysis** |  |  |  |  |  |  |  |  |  |  |
| 0854 Area1 | Coelophysis RIB | 61.66 | 21.01 | 84.09 | 20.31 | 817.82 | 348 | 1719.72 | 2.35 |  |
| 0854 Area2 | Coelophysis RIB | 182.18 | 61.66 | 229.67 | 74.61 | 821.10 | 413 | 1871.87 | 1.99 |  |
| 0854 Area3 | Coelophysis RIB | 70.82 | 21.53 | 114.06 | 27.91 | 687.57 | 246 | 1489.19 | 2.79 |  |
| 3888 Area1 | Coelophysis VRT | 181.00 | 77.92 | 212.87 | 82.54 | 605.95 | 196 | 1424.77 | 3.09 |  |
| 0854 Area4 | Coelophysis RIB | 61.28 | 26.74 | 82.37 | 29.32 | 828.47 | 346 | 1739.30 | 2.39 |  |
|  |  |  |  |  |  |  |  |  |  |  |
| **Herrerasaurus** | |  |  |  |  |  |  |  |  |  |
| 0331 Area1 | Herrerasaurus NSP | 272.06 | 63.15 | 294.86 | 58.64 | 556.81 | 176 | 1408.48 | 3.16 |  |
| 0331 Area2 | Herrerasaurus NSP | 116.47 | 24.75 | 140.77 | 30.21 | 590.48 | 182 | 1321.72 | 3.24 |  |
| 0331 Area3 | Herrerasaurus NSP | 325.80 | 114.38 | 285.24 | 67.60 | 603.12 | 227 | 1491.49 | 2.66 |  |
| 0333 Area1 | Herrerasaurus NSP | 145.67 | 39.57 | 163.53 | 42.72 | 702.14 | 271 | 1567.80 | 2.59 |  |
| 0333 Area2 | Herrerasaurus NSP | 166.53 | 63.35 | 147.75 | 33.42 | 723.08 | 302 | 1593.91 | 2.39 |  |
| 0333 Area3 | Herrerasaurus NSP | 286.33 | 54.30 | 289.21 | 54.72 | 627.98 | 240 | 1545.17 | 2.62 |  |
| 0333 Area4 | Herrerasaurus NSP | 329.94 | 57.88 | 273.68 | 45.19 | 657.44 | 242 | 1588.55 | 2.72 |  |
| 0333 Area6 | Herrerasaurus NSP | 230.85 | 55.48 | 218.20 | 46.19 | 641.62 | 221 | 1501.44 | 2.90 |  |
| 0334 Area1 | Herrerasaurus VRT | 257.54 | 62.99 | 240.75 | 48.53 | 688.65 | 277 | 1618.06 | 2.49 |  |
| 0516 Area1 | Herrerasaurus VRT | 110.58 | 37.79 | 107.93 | 39.66 | 757.78 | 262 | 1623.49 | 2.89 |  |
| 0516 Area2 | Herrerasaurus VRT | 155.86 | 31.03 | 154.33 | 33.29 | 769.58 | 277 | 1693.50 | 2.78 |  |
|  |  |  |  |  |  |  |  |  |  |  |
| **Sauropoda** |  |  |  |  |  |  |  |  |  |  |
| 0535 Area1 | Titanosauria | 212.29 | 40.85 | 226.16 | 45.22 | 649.44 | 203 | 1525.04 | 3.20 |  |
| 0535 Area2 | Titanosauria | 182.69 | 41.11 | 210.47 | 37.08 | 713.81 | 266 | 1638.09 | 2.68 |  |
| 0535 Area3 | Titanosauria | 285.61 | 78.13 | 315.47 | 65.91 | 583.08 | 191 | 1481.62 | 3.05 |  |
| 0535 Area4 | Titanosauria | 132.52 | 34.02 | 147.86 | 41.45 | 708.78 | 254 | 1565.42 | 2.79 |  |
| 0535 Area5 | Titanosauria | 197.99 | 51.94 | 186.22 | 47.35 | 651.02 | 220 | 1488.26 | 2.96 |  |
| 0535 Area6 | Titanosauria | 240.89 | 50.13 | 232.35 | 43.40 | 661.70 | 250 | 1555.74 | 2.65 |  |
| 0535 Area7 | Titanosauria | 93.24 | 27.98 | 108.64 | 30.74 | 720.93 | 273 | 1550.51 | 2.64 |  |
| 4927 Area1 | Diplodocidae FEM | 187.24 | 31.85 | 265.54 | 44.82 | 635.09 | 218 | 1535.73 | 2.91 |  |
| 4927 Area2 | Diplodocidae FEM | 189.13 | 69.43 | 151.57 | 47.91 | 671.24 | 268 | 1494.04 | 2.50 |  |
| 4105 Area1 | Titanosauria | 110.44 | 44.69 | 123.12 | 48.96 | 622.66 | 202 | 1368.44 | 3.08 |  |
| 0537 Area1 | Titanosauria | 126.85 | 59.68 | 128.79 | 37.72 | 695.90 | 241 | 1520.59 | 2.89 |  |
| 0993 Area1 | Titanosauria | 130.30 | 29.84 | 143.52 | 33.31 | 663.07 | 246 | 1469.66 | 2.70 |  |
|  |  |  |  |  |  |  |  |  |  |  |
| **Adeopapposaurus** | |  |  |  |  |  |  |  |  |  |
| 4257 Area1 | Adeopapposaurus NSP | 273.55 | 82.52 | 317.00 | 87.80 | 561.30 | 171 | 1439.60 | 3.28 |  |
| 4257 Area2 | Adeopapposaurus NSP | 107.74 | 46.26 | 100.43 | 39.59 | 763.30 | 288 | 1627.03 | 2.65 |  |
| 4257 Area3 | Adeopapposaurus NSP | 210.15 | 37.33 | 231.95 | 34.19 | 588.89 | 191 | 1409.72 | 3.08 |  |
| 4257 Area4 | Adeopapposaurus NSP | 136.98 | 39.88 | 183.82 | 22.98 | 587.53 | 165 | 1358.88 | 3.56 |  |
| 4257 Area5 | Adeopapposaurus NSP | 106.86 | 67.65 | 101.40 | 53.63 | 757.30 | 327 | 1615.99 | 2.32 |  |
|  |  |  |  |  |  |  |  |  |  |  |
| **Dinornis** |  |  |  |  |  |  |  |  |  |  |
| 0629 Area1 | Dinornis TMT | 185.19 | 46.43 | 187.35 | 47.69 | 605.46 | 196 | 1398.27 | 3.09 |  |
| 0629 Area2 | Dinornis TMT | 223.80 | 79.51 | 224.03 | 64.88 | 532.30 | 170 | 1288.63 | 3.13 |  |
| 0650 Area1 | Dinornis TMT | 147.05 | 33.64 | 155.08 | 36.25 | 598.02 | 196 | 1351.12 | 3.05 |  |
| 0650 Area2 | Dinornis TMT | 161.78 | 33.44 | 189.73 | 35.86 | 686.74 | 227 | 1563.21 | 3.03 |  |
| 0650 Area3 | Dinornis TMT | 446.06 | 82.06 | 416.41 | 78.09 | 469.85 | 122 | 1356.10 | 3.85 |  |
| 0650 Area4 | Dinornis TMT | 384.45 | 133.60 | 405.49 | 148.85 | 510.22 | 154 | 1425.93 | 3.31 |  |
|  |  |  |  |  |  |  |  |  |  |  |
| **Ornithischia** |  |  |  |  |  |  |  |  |  |  |
| 0004 Area1 | Ornithischia RIB | 303.36 | 77.62 | 253.97 | 58.99 | 616.71 | 208 | 1487.39 | 2.96 |  |
| 0004 Area2 | Ornithischia RIB | 361.34 | 97.76 | 390.58 | 87.30 | 523.37 | 161 | 1437.31 | 3.25 |  |
| 0004 Area3 | Ornithischia RIB | 310.11 | 78.84 | 337.29 | 74.85 | 522.22 | 174 | 1381.74 | 3.00 |  |
| 0004 Area4 | Ornithischia RIB | 349.83 | 84.93 | 328.02 | 57.86 | 445.13 | 114 | 1218.28 | 3.90 |  |
| 0005 Area1 | Ornithischia RIB | 190.46 | 57.93 | 180.80 | 39.93 | 564.18 | 192 | 1309.16 | 2.94 |  |
| 0005 Area3 | Ornithischia RIB | 388.49 | 87.64 | 352.70 | 68.59 | 468.80 | 122 | 1290.30 | 3.84 |  |
| 0006 Area1 | Ornithischia RIB | 531.60 | 140.40 | 565.38 | 156.24 | 449.96 | 154 | 1465.30 | 2.92 |  |
| 0006 Area2 | Ornithischia RIB | 211.47 | 56.45 | 219.97 | 46.07 | 596.71 | 227 | 1413.39 | 2.63 |  |
| 0006 Area3 | Ornithischia RIB | 331.27 | 69.90 | 325.70 | 58.80 | 584.14 | 199 | 1493.98 | 2.94 |  |
| 0185 Area1 | Hadrosauridae TEND | 288.43 | 158.20 | 203.87 | 98.65 | 642.87 | 216 | 1489.62 | 2.98 |  |
| 0185 Area2 | Hadrosauridae TEND | 497.70 | 135.85 | 395.97 | 80.49 | 533.35 | 187 | 1462.66 | 2.85 |  |
| 1038 Area1 | Triceratops HRN | 505.21 | 139.60 | 431.05 | 99.36 | 457.98 | 128 | 1347.01 | 3.58 |  |
|  |  |  |  |  |  |  |  |  |  |  |
| **Crurotarsi** |  |  |  |  |  |  |  |  |  |  |
| 2227 Area1 | Alligator TIB | 103.03 | 49.14 | 111.53 | 50.37 | 628.81 | 188 | 1369.15 | 3.34 |  |
| 2227 Area2 | Alligator TIB | 75.16 | 45.15 | 83.79 | 44.56 | 681.25 | 223 | 1446.30 | 3.05 |  |
| 2278 Area1 | Alligator TIB | 255.55 | 69.23 | 274.06 | 66.27 | 571.70 | 148 | 1417.46 | 3.86 |  |
| 4393 Area1 | Alligator TIB | 306.78 | 78.23 | 308.16 | 60.89 | 605.37 | 209 | 1518.90 | 2.90 |  |
| 4880 Area1 | Phytosaur VRT | 168.17 | 43.61 | 168.67 | 37.03 | 662.99 | 182 | 1494.66 | 3.64 |  |
| 4880 Area3 | Phytosaur VRT | 150.63 | 116.94 | 144.15 | 101.26 | 644.76 | 211 | 1433.66 | 3.06 |  |
| 4880 Area4 | Phytosaur VRT | 126.55 | 57.98 | 140.56 | 45.68 | 669.78 | 232 | 1480.12 | 2.89 |  |
|  |  |  |  |  |  |  |  |  |  |  |
| **Mammalia** |  |  |  |  |  |  |  |  |  |  |
| 4557 Area1 | Dasypus FEM | 260.66 | 85.49 | 259.44 | 79.26 | 534.54 | 159 | 1328.52 | 3.36 |  |
| 4557 Area2 | Dasypus FEM | 169.34 | 99.55 | 195.40 | 66.76 | 584.30 | 185 | 1364.01 | 3.16 |  |
| 4556 Area1 | Dasypus FEM | 170.57 | 91.88 | 124.15 | 59.81 | 636.14 | 210 | 1396.42 | 3.03 |  |
| 4766 Area1 | Didelphis ULN | 367.78 | 124.71 | 307.14 | 70.06 | 528.67 | 136 | 1364.48 | 3.89 |  |
| 4766 Area2 | Didelphis ULN | 321.66 | 82.93 | 301.90 | 73.55 | 552.36 | 175 | 1406.63 | 3.16 |  |
| 4775 Area1 | Capromys HUM | 372.42 | 111.93 | 220.35 | 70.12 | 665.03 | 212 | 1550.40 | 3.14 |  |
| 4764 Area1 | Antilocapra MTT | 204.06 | 48.98 | 244.11 | 49.17 | 644.99 | 184 | 1534.09 | 3.51 |  |
| 4764 Area2 | Antilocapra MTT | 174.30 | 56.55 | 215.77 | 57.31 | 649.15 | 184 | 1514.08 | 3.53 |  |
| 4764 Area3 | Antilocapra MTT | 168.83 | 58.80 | 203.70 | 65.19 | 640.78 | 187 | 1485.27 | 3.43 |  |
| 4764 Area4 | Antilocapra MTT | 206.92 | 71.84 | 205.72 | 42.14 | 682.92 | 198 | 1571.57 | 3.45 |  |
| 4755 Area1 | Canis TIB | 359.55 | 106.71 | 356.31 | 84.73 | 514.38 | 148 | 1385.08 | 3.48 |  |
| 4755 Area2 | Canis TIB | 231.67 | 57.93 | 288.61 | 53.95 | 515.55 | 154 | 1319.70 | 3.35 |  |
| 4757 Area1 | Canis TIB | 430.31 | 143.73 | 432.75 | 112.72 | 518.92 | 200 | 1470.58 | 2.59 |  |
| 4761 Area1 | Puma MTC | 191.53 | 70.66 | 182.60 | 46.09 | 663.55 | 195 | 1509.70 | 3.40 |  |
|  |  |  |  |  |  |  |  |  |  |  |
| **Lacertilia** |  |  |  |  |  |  |  |  |  |  |
| 616 Area 1 | Heloderma VRT | 230.65 | 111.62 | 243.71 | 106.47 | 638.65 | 177 | 1521.01 | 3.61 |  |
| 616 Area 2 | Heloderma VRT | 212.60 | 88.86 | 207.29 | 90.55 | 643.33 | 171 | 1493.95 | 3.76 |  |
| 0883 Area 1 | Tupinambis HUM | 417.48 | 119.24 | 339.35 | 116.16 | 520.49 | 173 | 1380.34 | 3.01 |  |
| 0883 Area 2 | Tupinambis HUM | 561.92 | 126.65 | 442.25 | 86.52 | 492.15 | 158 | 1426.56 | 3.11 |  |
| 4782 Area 2 | Tupinambis HUM | 380.61 | 80.79 | 314.61 | 55.00 | 540.11 | 170 | 1394.83 | 3.18 |  |
| 4781 Area 1 | Tarantola VRT | 236.99 | 67.24 | 217.62 | 51.91 | 662.90 | 176 | 1543.43 | 3.77 |  |

Each value is the total area, length or count of features in a square 200 µm^2^ area of thin-section.

^Abbreviations^:

Brs Branches

Can Canaliculi

FEM Femur

HUM Humerus

Lac Lacuna

Lgth Length

MTC Metacarpal

MTT Metatarsus

NSP Neural spine

Peri Perimeter

TEND Tendon

TBT Tibiotarsus

TIB Tibia

TMT Tarsometatarsus

ULN Ulna

VRT Vertebra
