# Supplementary material for: Correction: Bone Cells in Birds Show Exceptional Surface Area, a Characteristic Tracing Back to Saurischian Dinosaurs of the Late Triassic
Source: PLoS One. 2015 May 1;10(5):e0127373. doi: 10.1371/journal.pone.0127373 (PMC4416927; doi:10.1371/journal.pone.0127373)
Supplement: S1 Table — (DOC) [file pone.0127373.s001.doc]

| Sample | Description | Total | Largest | Total | Max. | Total | Total | Total | Av. |  |
| --- | --- | --- | --- | --- | --- | --- | --- | --- | --- | --- |
|  |  | Lac | Lac | Lac | Lac | Can | Can | Lac + | Can |  |
|  |  | Area | Area | Peri | Peri | Lgth | Br | Can Peri | Lgth |  |
|  |  |  |  |  |  |  |  |  |  |  |
|  |  |  |  |  |  |  |  |  |  |  |
| **Extant Neornithes** | |  |  |  |  |  |  |  |  |  |
| 4738 Area1 | Buteo HUM | 131.35 | 40.50 | 132.81 | 33.26 | 715.99 | 308 | 1564.79 | 2.32 |  |
| 0013 Area1 | Buteo FEM | 116.32 | 28.94 | 124.57 | 24.33 | 835.96 | 376 | 1796.49 | 2.22 |  |
| 0013 Area3 | Buteo FEM | 116.73 | 36.92 | 102.99 | 31.93 | 807.46 | 327 | 1717.91 | 2.47 |  |
| 4806 Area1 | Buteo TBT | 53.07 | 35.23 | 64.01 | 30.25 | 860.49 | 403 | 1784.99 | 2.14 |  |
| 4806 Area2 | Buteo TBT | 119.85 | 33.44 | 118.87 | 25.02 | 804.31 | 337 | 1727.48 | 2.39 |  |
| 4806 Area3 | Buteo TBT | 85.95 | 27.56 | 101.86 | 27.09 | 734.27 | 261 | 1570.40 | 2.81 |  |
| 0191 Area1 | Bubo ULN | 145.87 | 65.60 | 131.11 | 45.15 | 751.26 | 283 | 1633.62 | 2.65 |  |
| 4736 Area1 | Haliaeetus TMT | 130.92 | 35.36 | 144.55 | 37.86 | 768.61 | 302 | 1681.78 | 2.55 |  |
| 0193 Area1 | Cygnus TBT | 194.48 | 32.06 | 182.77 | 27.39 | 658.92 | 196 | 1500.60 | 3.36 |  |
| 4772 Area1 | Mergus TBT | 122.15 | 21.32 | 169.01 | 21.23 | 755.17 | 248 | 1679.36 | 3.05 |  |
| 4772 Area2 | Mergus TBT | 87.87 | 18.84 | 110.99 | 23.39 | 825.92 | 321 | 1762.83 | 2.57 |  |
|  |  |  |  |  |  |  |  |  |  |  |
| **Ornithomimidae &Tyrannosaurus** | |  |  |  |  |  |  |  |  |  |
| 0349 Area2 | Tyrannosaurus PHL | 164.23 | 78.02 | 160.50 | 81.88 | 710.99 | 258 | 1582.48 | 2.76 |  |
| 018 Area1 | Tyrannosaurus PHL | 198.39 | 55.07 | 177.78 | 46.49 | 727.26 | 265 | 1632.29 | 2.74 |  |
| 018 Area2 | Tyrannosaurus PHL | 284.54 | 44.33 | 248.67 | 37.30 | 664.73 | 256 | 1578.14 | 2.60 |  |
| 019 Area1 | Tyrannosaurus PHL | 189.59 | 57.83 | 196.89 | 60.67 | 698.46 | 277 | 1593.82 | 2.52 |  |
| 4434 Area2 | Ornithomimidae PHL | 83.96 | 18.05 | 104.44 | 20.40 | 696.91 | 251 | 1498.27 | 2.78 |  |
| 4434 Area3 | Ornithomimidae PHL | 117.50 | 38.14 | 127.30 | 37.64 | 702.87 | 269 | 1533.04 | 2.61 |  |
| 4825 Area1 | Ornithomimidae MTT | 113.82 | 39.57 | 139.35 | 43.06 | 790.35 | 352 | 1720.04 | 2.25 |  |
| 4825 Area2 | Ornithomimidae MTT | 242.31 | 73.73 | 246.98 | 55.71 | 776.86 | 397 | 1800.70 | 1.96 |  |
| 4825 Area3 | Ornithomimidae MTT | 151.86 | 51.18 | 165.99 | 35.82 | 738.68 | 303 | 1643.35 | 2.44 |  |
| 4825 Area4 | Ornithomimidae MTT | 127.01 | 50.31 | 135.21 | 33.64 | 816.93 | 370 | 1769.07 | 2.21 |  |
| 4825 Area5 | Ornithomimidae MTT | 162.44 | 43.46 | 163.21 | 34.43 | 736.32 | 287 | 1635.86 | 2.57 |  |
|  |  |  |  |  |  |  |  |  |  |  |
| **Coelophysis** |  |  |  |  |  |  |  |  |  |  |
| 0854 Area1 | Coelophysis RIB | 61.66 | 21.01 | 84.09 | 20.31 | 817.82 | 348 | 1719.72 | 2.35 |  |
| 0854 Area2 | Coelophysis RIB | 182.18 | 61.66 | 229.67 | 74.61 | 821.10 | 413 | 1871.87 | 1.99 |  |
| 0854 Area3 | Coelophysis RIB | 70.82 | 21.53 | 114.06 | 27.91 | 687.57 | 246 | 1489.19 | 2.79 |  |
| 3888 Area1 | Coelophysis VRT | 181.00 | 77.92 | 212.87 | 82.54 | 605.95 | 196 | 1424.77 | 3.09 |  |
| 0854 Area4 | Coelophysis RIB | 61.28 | 26.74 | 82.37 | 29.32 | 828.47 | 346 | 1739.30 | 2.39 |  |
|  |  |  |  |  |  |  |  |  |  |  |
| **Herrerasaurus** | |  |  |  |  |  |  |  |  |  |
| 0331 Area1 | Herrerasaurus NSP | 272.06 | 63.15 | 294.86 | 58.64 | 556.81 | 176 | 1408.48 | 3.16 |  |
| 0331 Area2 | Herrerasaurus NSP | 116.47 | 24.75 | 140.77 | 30.21 | 590.48 | 182 | 1321.72 | 3.24 |  |
| 0331 Area3 | Herrerasaurus NSP | 325.80 | 114.38 | 285.24 | 67.60 | 603.12 | 227 | 1491.49 | 2.66 |  |
| 0333 Area1 | Herrerasaurus NSP | 145.67 | 39.57 | 163.53 | 42.72 | 702.14 | 271 | 1567.80 | 2.59 |  |
| 0333 Area2 | Herrerasaurus NSP | 166.53 | 63.35 | 147.75 | 33.42 | 723.08 | 302 | 1593.91 | 2.39 |  |
| 0333 Area3 | Herrerasaurus NSP | 286.33 | 54.30 | 289.21 | 54.72 | 627.98 | 240 | 1545.17 | 2.62 |  |
| 0333 Area4 | Herrerasaurus NSP | 329.94 | 57.88 | 273.68 | 45.19 | 657.44 | 242 | 1588.55 | 2.72 |  |
| 0333 Area6 | Herrerasaurus NSP | 230.85 | 55.48 | 218.20 | 46.19 | 641.62 | 221 | 1501.44 | 2.90 |  |
| 0334 Area1 | Herrerasaurus VRT | 257.54 | 62.99 | 240.75 | 48.53 | 688.65 | 277 | 1618.06 | 2.49 |  |
| 0516 Area1 | Herrerasaurus VRT | 110.58 | 37.79 | 107.93 | 39.66 | 757.78 | 262 | 1623.49 | 2.89 |  |
| 0516 Area2 | Herrerasaurus VRT | 155.86 | 31.03 | 154.33 | 33.29 | 769.58 | 277 | 1693.50 | 2.78 |  |
|  |  |  |  |  |  |  |  |  |  |  |
| **Sauropoda** |  |  |  |  |  |  |  |  |  |  |
| 0535 Area1 | Titanosauria | 212.29 | 40.85 | 226.16 | 45.22 | 649.44 | 203 | 1525.04 | 3.20 |  |
| 0535 Area2 | Titanosauria | 182.69 | 41.11 | 210.47 | 37.08 | 713.81 | 266 | 1638.09 | 2.68 |  |
| 0535 Area3 | Titanosauria | 285.61 | 78.13 | 315.47 | 65.91 | 583.08 | 191 | 1481.62 | 3.05 |  |
| 0535 Area4 | Titanosauria | 132.52 | 34.02 | 147.86 | 41.45 | 708.78 | 254 | 1565.42 | 2.79 |  |
| 0535 Area5 | Titanosauria | 197.99 | 51.94 | 186.22 | 47.35 | 651.02 | 220 | 1488.26 | 2.96 |  |
| 0535 Area6 | Titanosauria | 240.89 | 50.13 | 232.35 | 43.40 | 661.70 | 250 | 1555.74 | 2.65 |  |
| 0535 Area7 | Titanosauria | 93.24 | 27.98 | 108.64 | 30.74 | 720.93 | 273 | 1550.51 | 2.64 |  |
| 4927 Area1 | Diplodocidae FEM | 187.24 | 31.85 | 265.54 | 44.82 | 635.09 | 218 | 1535.73 | 2.91 |  |
| 4927 Area2 | Diplodocidae FEM | 189.13 | 69.43 | 151.57 | 47.91 | 671.24 | 268 | 1494.04 | 2.50 |  |
| 4105 Area1 | Titanosauria | 110.44 | 44.69 | 123.12 | 48.96 | 622.66 | 202 | 1368.44 | 3.08 |  |
| 0537 Area1 | Titanosauria | 126.85 | 59.68 | 128.79 | 37.72 | 695.90 | 241 | 1520.59 | 2.89 |  |
| 0993 Area1 | Titanosauria | 130.30 | 29.84 | 143.52 | 33.31 | 663.07 | 246 | 1469.66 | 2.70 |  |
|  |  |  |  |  |  |  |  |  |  |  |
| **Adeopapposaurus** | |  |  |  |  |  |  |  |  |  |
| 4257 Area1 | Adeopapposaurus NSP | 273.55 | 82.52 | 317.00 | 87.80 | 561.30 | 171 | 1439.60 | 3.28 |  |
| 4257 Area2 | Adeopapposaurus NSP | 107.74 | 46.26 | 100.43 | 39.59 | 763.30 | 288 | 1627.03 | 2.65 |  |
| 4257 Area3 | Adeopapposaurus NSP | 210.15 | 37.33 | 231.95 | 34.19 | 588.89 | 191 | 1409.72 | 3.08 |  |
| 4257 Area4 | Adeopapposaurus NSP | 136.98 | 39.88 | 183.82 | 22.98 | 587.53 | 165 | 1358.88 | 3.56 |  |
| 4257 Area5 | Adeopapposaurus NSP | 106.86 | 67.65 | 101.40 | 53.63 | 757.30 | 327 | 1615.99 | 2.32 |  |
|  |  |  |  |  |  |  |  |  |  |  |
| **Dinornis** |  |  |  |  |  |  |  |  |  |  |
| 0629 Area1 | Dinornis TMT | 185.19 | 46.43 | 187.35 | 47.69 | 605.46 | 196 | 1398.27 | 3.09 |  |
| 0629 Area2 | Dinornis TMT | 223.80 | 79.51 | 224.03 | 64.88 | 532.30 | 170 | 1288.63 | 3.13 |  |
| 0650 Area1 | Dinornis TMT | 147.05 | 33.64 | 155.08 | 36.25 | 598.02 | 196 | 1351.12 | 3.05 |  |
| 0650 Area2 | Dinornis TMT | 161.78 | 33.44 | 189.73 | 35.86 | 686.74 | 227 | 1563.21 | 3.03 |  |
| 0650 Area3 | Dinornis TMT | 446.06 | 82.06 | 416.41 | 78.09 | 469.85 | 122 | 1356.10 | 3.85 |  |
| 0650 Area4 | Dinornis TMT | 384.45 | 133.60 | 405.49 | 148.85 | 510.22 | 154 | 1425.93 | 3.31 |  |
|  |  |  |  |  |  |  |  |  |  |  |
| **Ornithischia** |  |  |  |  |  |  |  |  |  |  |
| 0004 Area1 | Ornithischia RIB | 303.36 | 77.62 | 253.97 | 58.99 | 616.71 | 208 | 1487.39 | 2.96 |  |
| 0004 Area2 | Ornithischia RIB | 361.34 | 97.76 | 390.58 | 87.30 | 523.37 | 161 | 1437.31 | 3.25 |  |
| 0004 Area3 | Ornithischia RIB | 310.11 | 78.84 | 337.29 | 74.85 | 522.22 | 174 | 1381.74 | 3.00 |  |
| 0004 Area4 | Ornithischia RIB | 349.83 | 84.93 | 328.02 | 57.86 | 445.13 | 114 | 1218.28 | 3.90 |  |
| 0005 Area1 | Ornithischia RIB | 190.46 | 57.93 | 180.80 | 39.93 | 564.18 | 192 | 1309.16 | 2.94 |  |
| 0005 Area3 | Ornithischia RIB | 388.49 | 87.64 | 352.70 | 68.59 | 468.80 | 122 | 1290.30 | 3.84 |  |
| 0006 Area1 | Ornithischia RIB | 531.60 | 140.40 | 565.38 | 156.24 | 449.96 | 154 | 1465.30 | 2.92 |  |
| 0006 Area2 | Ornithischia RIB | 211.47 | 56.45 | 219.97 | 46.07 | 596.71 | 227 | 1413.39 | 2.63 |  |
| 0006 Area3 | Ornithischia RIB | 331.27 | 69.90 | 325.70 | 58.80 | 584.14 | 199 | 1493.98 | 2.94 |  |
| 0185 Area1 | Hadrosauridae TEND | 288.43 | 158.20 | 203.87 | 98.65 | 642.87 | 216 | 1489.62 | 2.98 |  |
| 0185 Area2 | Hadrosauridae TEND | 497.70 | 135.85 | 395.97 | 80.49 | 533.35 | 187 | 1462.66 | 2.85 |  |
| 1038 Area1 | Triceratops HRN | 505.21 | 139.60 | 431.05 | 99.36 | 457.98 | 128 | 1347.01 | 3.58 |  |
|  |  |  |  |  |  |  |  |  |  |  |
| **Crurotarsi** |  |  |  |  |  |  |  |  |  |  |
| 2227 Area1 | Alligator TIB | 103.03 | 49.14 | 111.53 | 50.37 | 628.81 | 188 | 1369.15 | 3.34 |  |
| 2227 Area2 | Alligator TIB | 75.16 | 45.15 | 83.79 | 44.56 | 681.25 | 223 | 1446.30 | 3.05 |  |
| 2278 Area1 | Alligator TIB | 255.55 | 69.23 | 274.06 | 66.27 | 571.70 | 148 | 1417.46 | 3.86 |  |
| 4393 Area1 | Alligator TIB | 306.78 | 78.23 | 308.16 | 60.89 | 605.37 | 209 | 1518.90 | 2.90 |  |
| 4880 Area1 | Phytosaur VRT | 168.17 | 43.61 | 168.67 | 37.03 | 662.99 | 182 | 1494.66 | 3.64 |  |
| 4880 Area3 | Phytosaur VRT | 150.63 | 116.94 | 144.15 | 101.26 | 644.76 | 211 | 1433.66 | 3.06 |  |
| 4880 Area4 | Phytosaur VRT | 126.55 | 57.98 | 140.56 | 45.68 | 669.78 | 232 | 1480.12 | 2.89 |  |
|  |  |  |  |  |  |  |  |  |  |  |
| **Mammalia** |  |  |  |  |  |  |  |  |  |  |
| 4557 Area1 | Dasypus FEM | 260.66 | 85.49 | 259.44 | 79.26 | 534.54 | 159 | 1328.52 | 3.36 |  |
| 4557 Area2 | Dasypus FEM | 169.34 | 99.55 | 195.40 | 66.76 | 584.30 | 185 | 1364.01 | 3.16 |  |
| 4556 Area1 | Dasypus FEM | 170.57 | 91.88 | 124.15 | 59.81 | 636.14 | 210 | 1396.42 | 3.03 |  |
| 4766 Area1 | Didelphis ULN | 367.78 | 124.71 | 307.14 | 70.06 | 528.67 | 136 | 1364.48 | 3.89 |  |
| 4766 Area2 | Didelphis ULN | 321.66 | 82.93 | 301.90 | 73.55 | 552.36 | 175 | 1406.63 | 3.16 |  |
| 4775 Area1 | Capromys HUM | 372.42 | 111.93 | 220.35 | 70.12 | 665.03 | 212 | 1550.40 | 3.14 |  |
| 4764 Area1 | Antilocapra MTT | 204.06 | 48.98 | 244.11 | 49.17 | 644.99 | 184 | 1534.09 | 3.51 |  |
| 4764 Area2 | Antilocapra MTT | 174.30 | 56.55 | 215.77 | 57.31 | 649.15 | 184 | 1514.08 | 3.53 |  |
| 4764 Area3 | Antilocapra MTT | 168.83 | 58.80 | 203.70 | 65.19 | 640.78 | 187 | 1485.27 | 3.43 |  |
| 4764 Area4 | Antilocapra MTT | 206.92 | 71.84 | 205.72 | 42.14 | 682.92 | 198 | 1571.57 | 3.45 |  |
| 4755 Area1 | Canis TIB | 359.55 | 106.71 | 356.31 | 84.73 | 514.38 | 148 | 1385.08 | 3.48 |  |
| 4755 Area2 | Canis TIB | 231.67 | 57.93 | 288.61 | 53.95 | 515.55 | 154 | 1319.70 | 3.35 |  |
| 4757 Area1 | Canis TIB | 430.31 | 143.73 | 432.75 | 112.72 | 518.92 | 200 | 1470.58 | 2.59 |  |
| 4761 Area1 | Puma MTC | 191.53 | 70.66 | 182.60 | 46.09 | 663.55 | 195 | 1509.70 | 3.40 |  |
|  |  |  |  |  |  |  |  |  |  |  |
| **Lacertilia** |  |  |  |  |  |  |  |  |  |  |
| 616 Area 1 | Heloderma VRT | 230.65 | 111.62 | 243.71 | 106.47 | 638.65 | 177 | 1521.01 | 3.61 |  |
| 616 Area 2 | Heloderma VRT | 212.60 | 88.86 | 207.29 | 90.55 | 643.33 | 171 | 1493.95 | 3.76 |  |
| 0883 Area 1 | Tupinambis HUM | 417.48 | 119.24 | 339.35 | 116.16 | 520.49 | 173 | 1380.34 | 3.01 |  |
| 0883 Area 2 | Tupinambis HUM | 561.92 | 126.65 | 442.25 | 86.52 | 492.15 | 158 | 1426.56 | 3.11 |  |
| 4782 Area 2 | Tupinambis HUM | 380.61 | 80.79 | 314.61 | 55.00 | 540.11 | 170 | 1394.83 | 3.18 |  |
| 4781 Area 1 | Tarantola VRT | 236.99 | 67.24 | 217.62 | 51.91 | 662.90 | 176 | 1543.43 | 3.77 |  |

Each value is from a 2025 µm^2^ area of thin-section.

Values are shown in µm or µm^2^.

^Abbreviations^:

Br Branch

Can Canaliculi

FEM Femur

HUM Humerus

Lac Lacuna

Lgth Length

MTC Metacarpal

MTT Metatarsus

NSP Neural spine

Peri Perimeter

TEND Tendon

TIB Tibia

TBT Tibiotarsus

ULN Ulna

VRT Vertebra
